# Multistaged endoscopic full-thickness resection using full-thickness resection device for large recurrent polyp: a case report

**DOI:** 10.1016/j.igie.2025.08.008

**Published:** 2025-08-22

**Authors:** Taylor Bowler, Natalie Wilson, Rahul Karna, Mohammad Bilal

**Affiliations:** 1 Department of Internal Medicine, University of Minnesota Medical Center, Minneapolis, Minnesota, USA; 2 Department of Gastroenterology, Hepatology and Nutrition, University of Minnesota Medical Center, Minneapolis, Minnesota, USA; 3 Division of Advanced Endoscopy, University of Colorado Anschutz Medical Campus, Aurora, Colorado, USA

## Abstract

Endoscopic full-thickness resection (EFTR) using the full-thickness resection device (FTRD) (Ovesco Endoscopy AG, Tübingen, Germany) is an endoscopic technique for management of recurrent polyps up to 30 mm in size. However, data are currently limited on the use of multistaged piecemeal EFTR using the FTRD for larger recurrent and fibrotic polyps. Herein, we present the case of an 85-year-old man referred to advanced endoscopy for a 60-mm recurrent polyp adjacent to the ileocecal valve. The decision was made to perform piecemeal EFTR using the FTRD in a multistaged fashion because of its large size, technically challenging location, and multiple recurrences despite use of different resection modalities. A total of 3 colonoscopies with EFTR using the FTRD were performed with successful removal of the recurrent polyp. Our case report demonstrates that multistaged EFTR using the FTRD can be safe and effective for large polyps in anatomically difficult locations not amenable to conventional resection modalities.

## Introduction

Several endoscopic techniques exist for the management of recurrent polyps after initial endoscopic resection. Endoscopic full-thickness resection (EFTR) using a full-thickness resection device (FTRD) (Ovesco Endoscopy AG, Tübingen, Germany) is used for resection of recurrent polyps; however, this technique is limited to polyps up to 30 mm in size. Data are currently limited on the use of multistaged piecemeal EFTR for the management of recurrent and fibrotic polyps larger than 30 mm. Herein, we describe a patient with a 60-mm persistent recurrent polyp who underwent successful polyp removal with multistaged piecemeal EFTR using the FTRD.

## Case

**Figure 1:**
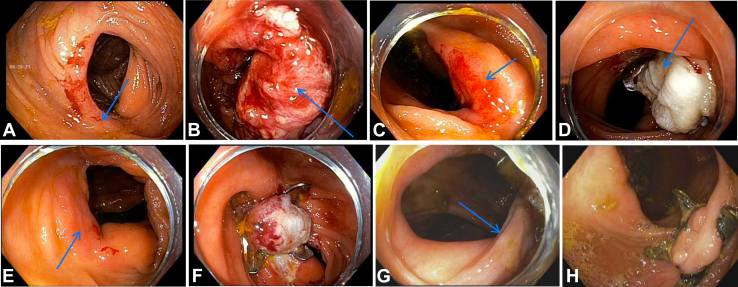
**A,** Recurrence of 60-mm polyp (*arrow*) on surveillance colonoscopy. **B,** Resection site (*arrow*) after use of the endoscopic-powered resection device (EndoRotor, Interscope Inc, Northbridge, Mass, USA). **C,** Recurrence of 60-mm polyp (*arrow*) on surveillance colonoscopy after use of the device. **D,** Resection site with clip (*arrow*) after first endoscopic full-thickness piecemeal resection. **E,** Residual polyp (*arrow*) before second endoscopic full-thickness piecemeal resection. **F,** Resection site after second endoscopic full-thickness piecemeal resection. **G,** Residual polyp (*arrow*) before third endoscopic full-thickness piecemeal resection. **H,** Resection site with no recurrence on surveillance colonoscopy after multistaged endoscopic full-thickness piecemeal resection.

An 85-year-old man with a medical history of prostate cancer, atrial fibrillation, congestive heart failure, hypertension, obstructive sleep apnea, tobacco use disorder with 26-pack years, and no relevant family or social history was referred for colonoscopy for evaluation of iron deficiency anemia. Findings on the physical examination were notable for a soft, nontender, and nondistended abdomen. On index colonoscopy, a 60-mm polyp was seen immediately distal to the ileocecal valve. Piecemeal cold-snare endoscopic mucosal resection (EMR) was performed by the referring endoscopist. Pathology demonstrated tubulovillous adenoma (TVA) with high-grade dysplasia (HGD). Surveillance colonoscopy at 6 months showed a 60-mm polyp in the same location suggestive of polyp recurrence (Fig. 1A). At this time, the patient was referred to advanced endoscopy for further management because he was deemed a poor surgical candidate. Resection attempts were initially made using the endoscopic-powered resection device (EndoRotor, Interscope Inc, Northbridge, Mass, USA) in combination with hot avulsion and hybrid argon plasma coagulation 6 months after index colonoscopy (Fig. 1B). Pathology redemonstrated TVA with HGD. On surveillance colonoscopy at 6 months, a 60-mm lesion was seen in the same location (Fig. 1C). After a multidisciplinary discussion, the decision was made to pursue multistaged EFTR with the FTRD in a piecemeal fashion because of the large size, technically challenging location, and recurrence despite previous use of different modalities. A total of 3 colonoscopies with EFTR using the FTRD were performed over 14 months with successful removal of the recurrent polyp (Fig. 1D-G). The over-the-scope clip placed with each FTRD had spontaneously fallen off before each reintervention. Pathology showed TVA with HGD after the first resection and TVA without HGD on the last 2 resections with negative deep margins on all specimens. The patient was discharged home safely on the same day as the procedures with recommendations to slowly advance his diet over the next 3 days. The patient tolerated the series of 3 procedures without any adverse events and did not require hospitalization after any procedure. Surveillance colonoscopy was completed at 12 months and showed no evidence of a residual or recurrent polyp (Fig. 1H). The patient and his family appreciated the minimally invasive approach because they wanted to avoid surgery with his advanced age and multiple comorbidities, but the patient did find the repeat colonoscopies and bowel preparations cumbersome.

## Discussion

Several options are available for management of large recurrent polyps, with each technique bearing its own advantages and limitations. Repeat EMR is the preferred modality for resection of recurrent lesions measuring less than 20 mm.^1^ Hybrid argon plasma coagulation and hot/cold avulsion often are used in conjunction with repeat EMR to reduce the risk of recurrence in atypical or larger lesions.^2^ Endoscopic submucosal dissection is an alternative approach used for resection of larger, superficial polyps not amenable to EMR, although this technique poses increased risk of perforation.^3^ The endoscopic-powered resection device is advantageous for resection of recurrent lesions with significant scarring, but can be difficult to maneuver in retroflexion and areas with significant looping.^4^ EFTR using the FTRD is another endoscopic technique reserved for fibrotic polyps in challenging anatomical locations; however, this technique is still limited to lesions measuring up to 30 mm due to the size of the FTRD diameter.^3^ If all conventional modalities are unsuccessful in managing recurrent and residual polyps, surgical options may be explored. However, surgery is associated with increased cost and risk of adverse events, especially in patients with multiple comorbidities.^5^

This report demonstrates that multistaged piecemeal EFTR using the FTRD may be a promising alternative when conventional modalities are unsuccessful in managing large recurrent or residual polyps. Furthermore, this intervention may offer a safe solution for large recurrent polyps in patients deemed poor surgical candidates due to medical comorbidities, as demonstrated in our patient. However, serial EFTR using the FTRD should only be considered after multidisciplinary discussion and shared decision-making with the patient, as piecemeal resection may be considered burdensome for both patient and physician due to the need for recurrent colonoscopies. Overall, this case demonstrates that serial EFTR using the FTRD can be useful in select patients with large recurrent polyps deemed challenging to manage with conventional endoscopic resection modalities.

## Guarantor

Mohammad Bilal, MD, FACP, FACG, Director of Endoscopy and Bariatric Endoscopy, Division of Advanced Endoscopy, University of Colorado Anschutz Medical Campus, 13001 East 17th Place, Aurora, CO 80045, USA. E-mail: billa17@hotmail.com.

## Patient consent

The patient in this article has given written informed consent to publication of their case details.

## Disclosure

The following authors disclosed financial relationships: M. Bilal: Consultant for Boston Scientific and Steris Endoscopy; paid speaker for Cook Endoscopy. All other authors disclosed no financial relationships.
